# Why and how unpredictability is implemented in aviation security – A first qualitative study

**DOI:** 10.1016/j.heliyon.2023.e13822

**Published:** 2023-02-17

**Authors:** Melina Zeballos, Carla Sophie Fumagalli, Signe Maria Ghelfi, Adrian Schwaninger

**Affiliations:** a School of Applied Psychology, University of Applied Sciences and Arts Northwestern Switzerland (FHNW), Riggenbachstrasse 16, 4600 Olten, Switzerland; b Center for Adaptive Security Research and Applications (CASRA), Thurgauerstrasse 39, 8050 Zurich, Switzerland; c Zurich State Police, Airport Division, Research and Development, Prime Center 1, 8058 Zurich Airport, Switzerland

**Keywords:** Unpredictability, Security measures, Insider threats, Aviation security, Qualitative research

## Abstract

In the past, aviation security regulations have mostly been reactive, responding to terrorist attacks by adding more stringent measures. In combination with the standardization of security control processes, this has resulted in a more predictable system that makes it easier to plan and execute acts of unlawful interference. The implementation of unpredictability, that is, variation of security controls, as a proactive approach could be beneficial for addressing risks coming from outside (terrorist attacks) and inside the system (insider threats). By conducting semi-structured interviews with security experts, this study explored why and how unpredictability is applied at airports. Results show that European airport stakeholders apply unpredictability measures for many reasons: To complement the security system, defeat the opponent, and improve human factor aspects of the security system. Unpredictability is applied at various locations, by different controlling authorities, to different target groups and application forms; nevertheless, the deployment is not evaluated systematically. Results also show how the variation of security controls can contribute to mitigating insider threats, for example, by reducing insider knowledge. Future research should focus on the evaluation of the deterrent effect of unpredictability to further give suggestions on how unpredictable measures should be realized to proactively address upcoming risks.

## 1 Introduction

Civil aviation has been in the focus of terrorism for more than 50 years (e.g., Refs. [1,2]). In response to terrorist attacks, aviation security measures have been continuously refined and improved (e.g., Ref. [3]). This was mainly based on minimizing the risk of known threats. Whenever a security-related incident occurred, weaknesses of the security system were identified, resulting in the adaptation of existing measures or the addition of new ones to improve aviation security (e.g., Ref. [4]). Indeed, up until today, most national and international standards and regulations have been *reactive* responses to past incidents. The problem with a uniquely reactive approach is that security is always one step behind as perpetrators may already evolve new threats to attack the system by exploiting another vulnerability (e.g., Ref. [5]). Unpredictability, that is, varying security measures to increase their deterrent effect and efficiency, allows a more *proactive* approach. First, it creates uncertainty regarding when, where, why, and how somebody is going to be controlled. This should make it more difficult for a perpetrator to plan and deploy an attack. Second, unpredictability has the potential to enhance security by distributing and making use of the available resources in a more effective and efficient way [6,7]. Third, it can be used to address insider threats, which has become more relevant since recent events such as the bomb explosion on Daallo Airlines Flight 159 [8]. Our study explored why and how unpredictability is applied at airports and whether insider threats are addressed with it. In the remaining introduction, we first briefly review the history of aviation security measures. We then discuss the concept of unpredictability and its relevance in addressing insider threats.

### 1.1 Brief history of aviation security measures

Civil aviation has been a target for terrorists since the early 1960s [2]. Before the 1960s, the aviation industry was confronted with technical challenges to safety in the skies [9]. The situation changed dramatically between 1968 and 1972 when a total of 326 cases of hijacking occurred [10], which at that time were mostly politically motivated. The Tokyo Convention (1963), the Hague Convention (1970), and the Montreal Convention (1970) resulted in standards and recommended practices for international civil aviation to prevent acts of unlawful interference against civil aviation [3]. They were first adopted by the International Civil Aviation Organziation (ICAO) Council in March 1974 and designated as Annex 17 to the Chicago Convention [11]. Moreover, the ICAO Aviation Security Manual (Doc 8973) was developed to assist member states in implementing Annex 17 by providing guidance on how to apply its standards and recommended practices [12]. Since then, Annex 17 and Doc 8973 are constantly being reviewed and amended considering new threats and technological developments [11,12]. The United States Federal Aviation Administration (FAA) introduced an anti-hijacking program in 1973 based on comprehensive screening of 100% of passengers and their luggage at airports [13]. It was the beginning of electronic security screening and the implementation of the magnetometer (today, the walk-through metal detector; WTMD) as standard practice for passenger screening [14,15]. In combination with this search, a given percentage of passengers (i.e., quota) were controlled more extensively by applying a full body pat down [14], which can be regarded as an early deployment of unpredictability.

As a result of the electronic security screening, the number of cases of hijacking reduced markedly [15]. During the 1980s, a shift from bargaining toward deliberate crashing was observed [16], which escalated in December 1988 with the tragedy of Lockerbie, in which an improvised explosive device (IED) was detonated in the hold of the aircraft of Pan Am Flight 103 [17]. The tragedy, which caused the death of all 259 passengers and crew on board and 11 people on the ground [17], gained substantial media presence [1,18]. As a result, standards were established for hold baggage screening (HBS), including the worldwide deployment of explosive detection systems for hold baggage [1]. The September 11, 2001 terrorist attack had an even higher impact, causing deaths of thousands of people (e.g., Ref. [3]). The terrorists used knives, which in those times could be brought on an aircraft, to take over the cockpit (cockpit doors were not protected) and to hijack multiple aircrafts, which were then used as weapons of mass destruction [18]. The post-9/11 era involved the immediate adaption of both law and security systems [19]; for example, knives were declared as prohibited articles, and protected cockpit doors were introduced. In addition, large investments were made in screening technology and training of personnel. These adaptions in security protocols and regulations were a direct consequence of the terrorist attack and resulted in stricter aviation security for all passengers (not risk-based; *reactive* regulation). In 2006, the liquid bomb plot of eight terrorists who planned to bomb seven airplanes with destinations to the U.S.A. and Canada was uncovered by British authorities before the terrorists could use the improvised liquid explosive devices [2,20]. This resulted in restrictions on liquids in hand luggage, and the development and deployment of new detection technology.

On one hand, the implementation and standardization of security measures and new processes led to a higher level of security; on the other hand, it resulted in an increased predictability of the security system [7]. Aviation security systems were tailored to react to past incidents and threats by the implementation and refinement of measures that were standardized (in order to reach security goals effectively; [21]). Two consequences of a reactive security approach were defined by Ref. ([5], p. 312): “(1) the reduction in the number of attacks from a current type of threat and (2) the creation of new threats” that are probably unknown by the system. In other words, as long as new standards and processes are created upon known incidents, the aviation security systems will remain one foot behind. Therefore, a more proactive paradigm that is based on unpredictability has become important [7].

### 1.2 Unpredictability in aviation security

The ICAO ([11], p. 3) defines unpredictability as: “The implementation of security measures in order to increase their deterrent effect and their efficiency, by applying them at irregular frequencies, different locations and/or with varying means, in accordance with a defined framework”. Ref. [7] points out that the implementation of unpredictability does not necessarily require additional resources, but instead, aims for a more effective and efficient application of security controls by varying them. This variation makes it difficult for an individual to predict whether he or she will be checked, and in what way. A situation of increased uncertainty is created intentionally, which generates the expectation that everybody can be controlled at any time; this, presumably, has a deterrent effect [22].

Unforeseeable security controls, that is, random searches, have been part of security systems for a longtime (e.g., full body pat down on a random basis since introduction of magnetometer/walk through metal detector; see Ref. [14]), but have recently received greater attention in form of explicitly formulated unpredictability concepts (e.g., the TSA's Playbook; [23]). A traditional security system follows a multi-layered approach ([24]; for an example see Ref. [25]) in which every layer represents a security measure working as a barrier to prevent acts of unlawful interference. However, every layer also involves (according to the swiss cheese model; [26]) loopholes representing vulnerabilities of the system. Such security gaps could result, for example, from predictability of the system or are simply not covered by security controls. Unpredictability could address these loopholes by varying the security controls.

As in theory, this proposition seems reasoned; several suggestions and regulations have already been made by international authorities: Some with mandatory character, for example, the random passenger control with explosive trace detection (ETD; [27]), and others with voluntary character, such as further deployment of unpredictability [11].

From an operational viewpoint, the intensification of “unpredictability” could have a positive impact on some key performance indicators. Supposing that fewer resources are spent on checking all passengers at the security checkpoint, and instead additional unpredictable checks take place elsewhere, a positive impact on efficiency (throughput), effectiveness (security), and passenger satisfaction is possible. However, first studies on perceived passenger experience have also shown that traditional security checks (everyone is screened equally) are perceived as fairer and safer than security checks based on random schedules (probability of being screened; [28]), but are also less convenient [29]. In a study by Ref. [30], it was shown that people perceive traditional security checks to be safer than randomized checks, irrespective of the percentage of people that are screened. Randomized security checks could consequently lead to a decreased perception of security.

The mechanisms of unpredictability need to be well understood in order to use its potential in the best way and to prevent undesired effects (i.e., reduced feeling of security). The deployment of unpredictability has to be cautiously planned and evaluated. Moreover, an unpredictable security system could also be beneficial for the mitigation of different threats—it is capable of not only addressing attacks “from outside,” as quoted above, but also threats that come from within the airport security system [31].

### 1.3 Insider threats

In the recent past, it was suspected that “insiders” played an increasingly important role in executing terrorist attacks [18,32]. The term “insider” refers to presently or previously authorized system users “who have legitimate access to sensitive/confidential material, and they may know the vulnerabilities of the deployed systems and business processes” (Ref. [33], p. 2). Thus, an “insider threat” is one or more individuals that have access to relevant infrastructure and/or insider knowledge which allows them to exploit vulnerabilities of the system and to cause harm to the organization [25,33].

Specifically, in the field of information security (IS), the insider threat has been widely investigated (e.g., Refs. [33,34]) since surveys showed that 27% of all cybercrime incidents are suspected to be committed by insiders [33,35]. The scientific discourse distinguishes between the malicious and the unintentional non-malicious insider threat. Both cause harm to the organization, but only the malicious insider acts intentionally [34]. The operation of an airport requires resources of thousands of employees, whereby some of them have access to sensitive security areas or work around and in the aircraft. In consequence, all airport employees with access to sensitive security areas and/or knowledge about relevant security processes can theoretically become an insider threat (e.g., Ref. [36]). Taking a closer look, different forms of *malicious insider* involvement are conceivable: An employee becomes radical after employment or is being instrumentalized by somebody outside the airport system. Furthermore, it is also reasonable to consider that an airport worker could accept bribes in order to pass a bag (containing restricted items) through security check without knowing that they are facilitating the placing of a bomb onboard the aircraft [18]. Therefore, typical activities of a malicious insider include “spying, release of information, sabotage, corruption, impersonation, theft, smuggling, and terrorist attacks” (Ref. [25], p. 2). In February 2016, a bomb exploded onboard Daallo Airlines Flight 159 from Mogadishu to Djibouti (e.g., Ref. [8]), almost killing passengers and crew. After passing the security check, the suicide bomber was passed a laptop-like device by at least one airport employee [8]. Further, the bomb explosion of the Metrojet Flight KGL9268 above Sinai is suspected to be facilitated by an insider working at the Sharm el-Sheikh International Airport (e.g., Ref. [18]).

The *unintentional non-malicious insider* is also critical since the individual “has no malicious intent associated with his/her action (or inaction),” “which caused harm or substantially increased the probability of future serious harm to the confidentiality, integrity, or availability of the organization's information […]” (Ref. [37], p. 3) or security. Potential incidents may include accidental disclosures of sensitive information or data as well as social engineering [37] which involves an outsider that tries to acquire information or data through an insider, for example, to gain access to restrictive areas or to plan an attack.

To the aviation industry, insider threats are seen as a growing problem that need the establishment of countermeasures [31,35,38]. In the context of a highly regulated security system acting mainly in a reactive way upon past incidents, insider threats seem crucial—precisely because the insider knows about potential security gaps due to the proximity in daily operations and could use this information either in a malicious or non-malicious way. Specifically, the predictability resulting from standardized application of security measures [7] makes the system more vulnerable, which is why insider threats need to be addressed proactively with new security concepts such as unpredictability.

In theory, the implementation of unpredictability within measures of a security system seems logical and straightforward. However, as there is often a gap between theory and application, and so far most studies on unpredictability have been conducted in a lab setting [22,[28], [29], [30]], it is still unclear why and how unpredictability is applied at airports and whether it is used to address insider threats. This leads to the present study and the corresponding research questions.

### 1.4 Present study

The present study addresses three research questions: 1. Why are unpredictable measures implemented at airports? 2. How are unpredictability measures actually applied at airports? 3. Can unpredictability contribute to mitigate insider threats according to practitioners? To consider different perspectives of stakeholders, we choose a qualitative research method with a semi-structured interview study design [39].

## 2 Methods

We conducted interviews with experts and executed on-site visits at airports to address the research questions. We defined an expert as a person that has expertise and/or privileged knowledge in the field of airport security's practice and is willing to disclose it following the definition of Ref. ([40], p. 98ff). To achieve a purposeful sampling [41], we aimed at getting experts from at least one appropriate national authority that regulates aviation security measures, at least one police organization operating at a large airport, at least one expert from an airline, and several airport security managers including large and small airports. Based on the network contacts of the 3rd and the last author and their organizations, we contacted 22 experts from different European countries by email through which we briefly presented the research project and asked for a confidential personal exchange. Eighteen experts responded by email and requested more information. We then spoke to them by telephone to provide them more detailed information about the study. Finally, after internal consultations, eight experts agreed to participate in the semi-structed interviews and on-site visits at the airport. Informed consent was obtained from all the participants. Table 1 lists the type of experts who participated in our study.

**Table 1:** Experts.

Expert	Country	Stakeholder group
1	A	Appropriate national authority
2	A	Police organization
3	A	Airport security management (large airport)
4	B	Airline security management (small airport 1)
5	B	Airport security management (small airport 1)
6	B	Airport security management (small airport 2)
7	B	Airport security management (small airport 3)
8	B	Airport security management (small airport 4)

Note. “Large” and “small” refer to the airport size categories of the Airports Council International (ACI), where large size airport means more than 25 million passengers per year, and small means less than 5 million passengers per year.

We developed an interview guideline for semi-structured interviews based on recommendations by Refs. [39,42]. The final interview guideline, including the interview questions, is provided in Appendix A. The interviews were conducted on-site at the airport and lasted between 60 and 90 min. One interviewer led the interview, and the second interviewer made an interview protocol. After a short introduction, including information about the project, the procedure of the interview was explained, and anonymity was guaranteed. After participants provided written informed consent, the interview started with general questions about the concept of unpredictability. In the second part, the experts were asked to brainstorm about the application of unpredictability in general. The third part was dedicated to collecting data of applied unpredictability measures at the airport without specific implementation details*.* In the fourth part, we asked how the stated measures were concretely implemented by using a question list that included questions about what happens where, when, how, why, who is responsible, and what the lessons are. In the fifth part, we asked how unpredictability measures are used to address insider threats*.* In the last part, the experts were asked about further remarks and future prospects of unpredictability (see Table 2).

**Table 2:** Covered topics of the interview guideline.

Section	Topic	Example questions
1	Concept of unpredictability	What do you [as an appropriate national authority/police organization/airline security manager/airport security manager] understand by the term «unpredictability»?
2	Application in general	What role/value does unpredictability play/have in general when applying/realizing security measures at the airport?
3	Overview of applied measures	What measures are already in use that you think have an unpredictability component?
4	Implementation of applied measures	How was this measure concretely implemented? Why was this measure concretely implemented?
5	Contribution to insider threats	How does your organization address the issue of insider threats?
6	Future prospects of unpredictability	How do you assess the future development of unpredictability measures?

Note. Interview topics defined the focus of the study and were covered in all interviews. However, the sequence of the topics was not strictly followed as is usual for semi-structured interviews [43].

To verify and complement the information obtained in the interviews, the two interviewers then conducted on-site observations with the interviewed experts for 15–90 min (at all except one airport). The questions and notes of the interview that was conducted before served as a guideline, and notes were taken by both interviewers. After the on-site observations, the interview protocol was finalized by the second interviewer and complemented by observational information before being reviewed by the lead interviewer. The on-site observations and interviews provided consistent results. In the few cases where we thought to have found discrepancies, we clarified them with the interviewed experts. The protocols were then sent to the experts who could request adjustments when they did not agree with certain statements, and they could request deletions when statements were too security sensitive.

To analyze data of the final protocols, we employed an inductive approach using content analysis [44] and MAXQDA Version 20.0.2 to develop the coding scheme. The two interviewers coded one interview separately and discussed discrepancies to further develop the coding scheme. Subsequently, all interviews were coded by both interviewers separately. We calculated the intercoder reliability (ICR) as suggested by Ref. [45]. We obtained a Cohen's Kappa of .80, which is regarded as good [46].

## 3 Results

The findings of the study are presented in this section following the recommendations of Ref. [47]. We will first look at why unpredictability is being applied [finding 1] before presenting the list of applied security measures [finding 2]. We further look at the varying elements of the identified security measures in more detail [finding 3] before addressing the potential of unpredictability to counter insider threats [finding 4]. At the end of this results section, we present three perspectives on how the mentioned measures are and could be integrated into a security system [finding 5].

### 3.1 Finding 1: Reasons for applying unpredictability

While analyzing the data, we first extracted all the reasons for applying unpredictability by using an inductive approach. Second, we summarized them into senseful units to reduce the data and refined the categories. In a subsequent step, we built four main categories, which allowed us to cluster the subcategories. Table 3 lists the different reasons ordered by how often they were mentioned.

**Table 3:** Main and subcategories of reasons for applying unpredictability measures.

Main category	Description of the main category	Subcategory	Description of the subcategory
Security system focused (46%)	Unpredictability is applied because it is mandated by (inter)national regulations and/or is seen as a proactive approach complementing baseline security measures.	Regulation (*n* = 9)	Unpredictability measures (e.g., random inspections of passengers, staff, and items being screened) are mandated by international and national regulations. Compliance with these regulations is important and therefore a reason for applying unpredictability measures.
		Closing gaps (*n* = 8)	A specific vulnerability of the system (i.e., a security gap) can be addressed by random checks (i.e., with unpredictable measures) that cannot be covered within the regular security measures. For example, a small size airport does not necessarily have explosive detection dogs (EDD) in use. This lack can be addressed with additional explosive trace detection (ETD) usage, which are carried out randomly on passengers and staff.
		Quality control (*n* = 4)	Unpredictability measures and tests can be used for quality control purposes to ensure that security measures are conducted effectively and provide feedback to employees on their performance.
		Proactive security (*n* = 1)	Unpredictability is seen as a proactive approach that complements baseline security measures that have been implemented as reactions to incidents.
Opponent focused (31%)	Unpredictability is applied to defeat the opponent of the security system through deterrence, confusion, and by impairing planning and collaboration.	Deterrence (*n* = 6)	The irregularity of controls can lead to a state of uncertainty for persons with malicious intentions in information gathering, in the planning of a criminal activity, and in the criminal act itself. It is assumed that the implementation of unpredictability and its appropriate communication to passengers and staff increases the deterrent effect of the security system.
		Confusion of the opponent (*n* = 5)	The irregularity of applying security measures aims at confusing potential opponents who are presumably observing security processes to identify regularities, vulnerabilities, and weaknesses.
		Prevention of planning and collaboration (*n* = 2)	If security measures are conducted in an unpredictable way, planning an attack becomes difficult. Moreover, an act of unlawful interference involving different persons from in and/or outside the system (e.g., a screener working at a checkpoint and a passenger with a bomb in carry-on baggage) becomes difficult.
		Visibility of security (*n* = 2)	Overt security measures (e.g., patrolling police officers) increase the visibility of security for passengers, staff, and visitors of an airport.
Human factors focused (23%)	Unpredictability is applied to enhance human factors by increasing awareness, training, and motivation of security staff.	Security awareness (*n* = 5)	Unpredictability measures can increase security awareness because security employees are more often confronted with the fact that something could happen. Therefore, it is assumed that random tests, for example, raise security awareness.
		Training (*n* = 3)	Some procedures, which are rarely carried out due to operational reasons, can be trained by applying them at an irregular frequency.
		Flexibility (*n* = 2)	Implementation of unpredictability measures can increase the cognitive flexibility of security employees as workflows and processes vary more.
		Variety/diversification (*n* = 1)	Boredom and monotony have a negative impact on work motivation and performance. Unpredictability measures reduce boredom and monotony by introducing variety in security processes, which could result in better work motivation and performance.

Note. The absolute number of statements was n = 48. Statements were included when they were related to a reason for application of unpredictability (either in combination to a specific measure or in general). Whenever a specific reason was mentioned twice within a measure or within an interview, only one statement was counted. This occurred once. The percentages in brackets refer to the number of statements of the main category relative to all statements.

### 3.2 Findings 2 and 3: Unpredictability measures and their varying elements

The analysis of the interview protocols resulted in ten unpredictability measures. They are listed in Table 4 ordered by the locations where they are applied. All were mentioned explicitly as unpredictability measures and described in detail. Other security measures, for example, air marshals and explosive detection dogs, were also mentioned but not explained in detail and therefore not included in Table 4. Subsequently, we looked at the varying elements of the identified security measures (Table 4). As we addressed specific questions within a specific unpredictable security measure in the interview (when, where, what, who), a more deductive approach was applied in the analysis. We chose to categorize the varying elements according to the interview guideline which resulted in four categories: Time of control, location of control, type of control, and controlling authority.

**Table 4:** Unpredictability measures ordered by locations where they are applied.

Where	Unpredictability measure	Short description of measure	Who	Target group	Variation/Randomization factor	Overt/covert application
In- & outside perimeter	Patrol activity	Patrols are executed in an unpredictable manner, whereby time entries and journals help to monitor the variation (the measure is often combined with “Badge/identity check”).	Airport security staff; security provider; police	Passengers/visitors; airport staff	Location of control Time of control Type of control	Overt Covert
Entire airport site	Badge/identity check	Random, irregular, and unexpected examination of an employee's identity and access rights for a certain area at the airport (without a quota; the measure is often combined with “Patrol activity”). This includes verifying that a badge/ID card is still valid.	Airport security staff; security provider; police	Airport staff	Location of control Time of control	Not mentioned
	Quality checks	Quality checks include the verification of compliance with security regulations/instructions among employees. These checks are not announced but defined by a system for random, uniform deployment.	Airport security staff; security provider; Airport security management	Airport staff	Location of control Time of control Type of control Controlling authority	Overt Covert
Gates of goods delivery	Quota-based checks on goods	Quota-based application of checks on goods delivered by unknown suppliers. The randomness is defined by a quota algorithm.	Airport security staff	Supplier (unknown); Airport staff	Time of control Type of control	Not mentioned
Entire airport site; mainly at the security checkpoint	Switch of workplaces	Working stations must be switched unexpectedly (team/individual A switches workplace with a team/individual B). The randomness is carried out from time to time (not structured or constantly).	Airport security management	Airport staff	Location of control Time of control	Covert
Security checkpoint	Quota-based ETD checks	Quota-based application of explosive trace detection (ETD) tests on passengers or baggage at the security checkpoint. The quota is given by the authority and applied by an algorithm.	Airport security staff	Passengers; Airport staff; Crews	Time of control	Not mentioned
	Assignment to security lanes	Random assignment to a specific security control lane at the end of the passenger queuing. The assignment is based on capacity of the security lanes (efficiency).	Airport security staff	Passengers; Airport security staff	Location of control	Covert
	Additional checks on passengers	Additional controls (e.g., ETD, pat down) are carried out at the discretion of security employees. The selection of passengers can be risk-based (e.g., a passenger behaves suspiciously) or random.	Airport security staff	Passengers	Time of control Type of control	Covert
Airside; at the gate	Match of ID/boarding card	The measure includes the comparison of the personal identification document (ID) and the boarding pass for intra-Schengen flights. The flights are selected randomly, with an annual event-based number of executions.	Ground handling staff; airline	Passengers	Time of control	Not mentioned
	Security controls airside (abroad)	Various measures are commissioned unannounced to security providers at foreign airports which do not meet the airline's quality standards (e.g., additional ETD checks on passengers, observation, repeated X-ray screening). The frequency of the measure depends on the relationship of trust between airline and security provider.	Security provider (abroad); airline	Passengers; Security provider (abroad)	Time of control Type of control Controlling authority	Overt Covert

In our data collection, we also examined whether unpredictability measures are evaluated. According to our data, measures were usually not systematically evaluated, which is why we do not report them in Table 4. However, one measure (assignment to security lanes) was evaluated in terms of its operational efficiency, and another measure (patrol activity) in terms of the variation of the measure carried out by employees.

### 3.3 Finding 4: Unpredictability's contribution to mitigate insider threats

As shown in Table 4, several unpredictability measures target groups that have insider knowledge (airport staff, crew members, and suppliers). To examine this aspect in more detail, we analyzed our data specifically regarding insider threats based on our coding scheme. This resulted in the following potentials:

*Reduced insider knowledge.* Compared to outsiders, the experts mentioned in the interviews that insiders have the advantage of being part of the security system and therefore have important knowledge on how the security system operates. This is a risk, in particular, if security processes and procedures are standardized and predictable. By varying security procedures and processes using unpredictability, a non-transparency of the system is intended in which the value of insider knowledge is reduced. Moreover, unpredictable and varying security procedures and processes make it difficult to plan and execute an attack.

*Reduced insider impact.* The experts pointed out that unpredictability has the power to reduce individual impact on the security control process due to randomization. For example, algorithms that control the random allocation of passengers and bags to a screening operator (e.g., multiplexing in combination with remote screening; [48,49]), serve as a preventive structure of the system because it neither allows influence on the controlled passenger and bag nor takes advantage of it.

*Increased deterrence.* According to the experts, randomization has the additional benefit of deterring insiders. When security procedures and processes are randomized, the probability of success of an act of unlawful interference is reduced and could therefore discourage insider actions.

*Increased flexibility.* The interviews have shown that unpredictability has the potential to require greater adaptability and flexibility from airport staff in performance of their duties which might reduce monotony and boredom at work. When procedures, processes, and team constellations vary, employees potentially experience variety and are challenged.

*Increased security awareness.* The interviewed experts stated that unpredictability helps to raise and maintain security awareness among employees and enables them to remain observant. Conducting unpredictability measures, as described in Table 4, could increase and maintain awareness of the existence of insider threats among employees, and therefore, security controls targeting employees are necessary and are being conducted. Due to increased awareness regarding insider threats, employees would also observe each other more closely and possibly discover unusual actions.

### 3.4 Finding 5: Perspectives on the application of unpredictability

Overall, three perspectives of how unpredictability can be integrated into an existing security system were identified inductively from the interview data.

*Unpredictability as a variant of regular security measures (1)*. In this perspective, unpredictability measures are not seen as self-contained or autonomous. Unpredictability, respectively the application of randomness, is only a method of applying and varying an existing security measure. For example, control areas are provided and randomness is integrated in the process of checks (sequence, time, and frequency). This application of unpredictability is reasonable where no seamless control is possible (e.g., patrol activity; see Table 4).

*Unpredictability in addition to regular security measures (2)*. Unpredictability is most often described as an additional measure to the regular security measures. In this perspective, unpredictability primarily serves to close security gaps by simply running on top of conventional measures. Unpredictability is seen as an ideal complement to a “predictable” security control process. Wherever regulatory provisions are not enough, unpredictability can close security gaps by simply adding an additional measure (e.g., security controls airside, see Table 4).

*Unpredictability as a substitute of regular security measures (3)*. A third perspective on unpredictability is currently not being applied but could be promising in the future (after proof of concept). In the interests of economic efficiency, unpredictability should also replace previous conventional measures by occasional, targeted unpredictability measures (e.g., quota-based checks on goods; see Table 4). Furthermore, from this perspective, the application of unpredictability could be risk-based.

## 4 Discussion

In the past, aviation security regulations and measures have mostly been reactive responses to terrorist attacks. In the last decade, a more proactive approach based on unpredictability has gained attention [6,7,22]. It involves varying security measures to increase their deterrent effect and their efficiency. In our study, we investigated why and how unpredictability is implemented at airports. In the following sections, we first discuss our findings. We then address limitations and further research before we finish with the conclusion.

### 4.1 Discussion of findings

Finding 1: The identified reasons for practicing unpredictable measures can be broken down to three main foci: Security system focused, opponent focused, and human factors focused reasons. The security system focus was most often mentioned. From this perspective, unpredictability is applied because it is mandated by (inter)national regulations and/or is seen as a proactive approach that is an extremely useful complement to baseline security measures. International [11], European (e.g., Refs. [27,50]), and several national regulations already mandate some measures (e.g., the random application of ETDs; [27]) or recommend to further apply unpredictability (e.g., Ref. [11]). Security experts consider unpredictability as a reasonable way to address specific vulnerabilities of the security system (i.e., close security gaps) by varying them. From the second perspective, unpredictability is applied to defeat opponents of the security system by increasing deterrence, creating confusion, and impairing planning and collaboration of persons with malicious intents. This perspective matches well with previous publications on the benefits of unpredictability [7,22]. Interestingly, we also found that unpredictability is applied to improve human factor aspects of the security system. Unpredictable variation of security controls is seen as a possibility to train the staff in executing security controls (or rarely applied security measures). Moreover, applying unpredictability is also regarded as being useful for increasing and maintaining security awareness in practice. As security awareness is learned through practical experience [51], an unexpected rigorous application of measures could transmit that something could happen any time.

Considering the multi-layered approach to security [24] in which every security measure works as a barrier to prevent acts of unlawful interference, the security aware human operator, who applies security measures in a less predictable way while at the same time being mindful toward unusual behavior of passengers and employees, could function as an additional human barrier (i.e., a security layer; [52]) and further strengthen security (by enhancing security decisions; [21]). From our perspective (and following Ref. [7]), the security culture at airports seems crucial when applying unpredictability. Airport staff should be trained and encouraged to report relevant security observations (e.g., Refs. [7,25]; for example, within an anonymous staff reporting system; [8]) and to apply unpredictability on their own within their restrictions.

Finding 2: Unpredictability is applied through different security measures, which vary in terms of form of implementation and location. Measures are applied land- and airside, focusing on the security checkpoint. The landside area is seen as vulnerable [53], especially since the attacks at Brussels Airport in 2016, where suicide bombers detonated their bombs in the departure hall area. Immediate reactions followed, and landside security measures were intensified; however, the bigger and more expensive area to protect is airport security [54]. In that case, unpredictability could be useful because limited resources could be distributed in a more effective way by varying security presence. For example, patrolling explosive detection dogs in combination with camera surveillance can be used to identify a person that reacts in an unusual manner to the unexpected presence of such patrols (compared to other people).

Looking at the overall application of unpredictability, it is notable that mostly defined algorithms are used to achieve a randomization for security controls of a target group (e.g., ETD-checks). This is regarded reasonable since humans are rather weak at generating randomness manually [55] and fall easily into predictable patterns [56]. In some cases, the execution of measures is, however, not structured nor planned but rather a side effect of operations (e.g., switch of workplaces). Which is why it is not surprising that unpredictability measures were not evaluated in terms of their effectiveness. It is therefore quite difficult to build up practical knowledge on how a measure should be applied (i.e., varied) to achieve the best effect under prevailing conditions without systematic evaluation. However, it must be considered that there is still limited knowledge about the concept of unpredictability, which makes it difficult to assess.

Finding 3: An unpredictable security measure typically includes a variable that is varied and/or randomized. Among the collected unpredictability measures, this variable was most often the time of control. The variation of the time when a control takes place is probably the easiest form of implementing unpredictability within a security system. It allows the entity to take into consideration the regular operational processes at the airport such as flight schedules, staff resources, and other daily business. Therefore, an entity can identify several possible time slots during business hours and (randomly) deploy the unpredictable measures, for example, quality checks. As long as the planning cycle for the deployment is changed from time to time and does not follow an easy-to-observe logic, this remains an adequate way to sustain an amount of unpredictability. Results showed that also the location and type of security control can be varied. Depending on the characteristics of the airport (e.g., layout and infrastructure; [57]) different types of implementations are possible. Specifically, smaller airports tend to be restricted in terms of adequate locations. For example, the assignment of passengers to different security lanes is not extremely effective if there is only a small number of lanes. Nevertheless, some measures can also be varied regarding location at smaller airports, for example, badge checking. The variation of the type of security controls also depends on infrastructure as well as on available resources (e.g., availability of explosive detection dogs). A remarkably interesting approach in implementing unpredictability is the variation of the controlling authorities (as it is done in quality checks; see Table 4), for example, by involving the local police or the appropriate national authority as an additional control entity. In our view, it is a promising approach as it contains not only the (observable) change in the authority but also more sublime aspects of unpredictability. In other words, two authorities will conduct the same measure to some extent in a different way due to organizational culture, training [58], and so forth. On the other hand, as pointed out by one of the reviewers, mixing multiple authorities in the same duties could create confusion and conflict that would burden the security efficiency and effectiveness.

Furthermore, the implementation should also consider an optimal balance between overt and covert applications as well as a good frequency of deployment. Extremely frequent deployment could lead to a habituation effect that could have a negative impact on deterrence and again make it more predictable, whereas an extremely infrequent use could lead to the measure not being present enough to be effective. Further, the implementation of overt and covert applications seems crucial, as many covert measures could decrease their deterrent effect (i.e., security seems not to be present), whereas many overt measures could also have an impact on perceived security of passengers (i.e., passengers do not feel safe when visible security guards are everywhere at the airport), and therefore affect the passenger experience negatively. Further, it must be noted that covert application of measures (e.g., switching of workplaces; see Table 4) lacks the possibility to evaluate them in terms of their deterrent effect because the measures are simply not visible to the target group. There are, however, mitigating factors for insider threats as discussed in the subsequent section.

Finding 4: Unpredictable measures with focus on airport and security employees can contribute to mitigating a potential insider threat. Results showed that different security measures already relate to unpredictability by variation and unpredictable changes, for example, of their workplace station. Specifically, for small size airports, where work schedules are more predictable than at larger airports, a switch of workplace appears to be an interesting approach. When looking at both types of insider threats, it seems that most unpredictability measures affect both the malicious and non-malicious insider. The implementation of unpredictability measures also supports a system of non-transparency [7], consequently, making it more difficult to build up knowledge because patterns of operations and processes are missing due to variation. Furthermore, unpredictability could cause lack of predictive capabilities (e.g., when planning to conduct an attack) when the opponent is aware of the variation and thereby deterred.

However, findings suggest that unpredictability also contributes to increasing and maintaining security awareness among airport employees and potentially creates a positive impact on the security culture: A security culture in which everyone is attentive and vigilant concerning possible threats is more sensitive toward blind spots. Hence, the concept of unpredictability could be considered in relation to programs for preventing insider threats [31].

Finding 5: We found three different perspectives on how unpredictability measures could be implemented into existing security concepts: regular security measures could be varied (1), added with unpredictability measures (2), or replaced by such measures (3). While a simple variation of a regular measure does not require any additional human resources, these are required for additional security measures. Both perspectives, however, promise a potential increase in security when applied in a targeted manner. From an economic point of view, the third perspective is promising as human resources would be reduced, throughput increased, and the passenger experience could be impacted positively when a substantial number of passengers can be categorized as low risk. However, this could also result in reduced security perception of passengers [30] when not communicating appropriately. Furthermore, security could be compromised by a reduced density of controls, which is why this option has not yet been applied. Our findings show that, currently, most unpredictability measures are regarded as variant or as an addition to existing security measures. Unpredictability is presently not replacing regular security measures; however, this approach could become relevant in the future. If it is possible that high-risk groups can be identified and differentiated more validly from low-risk groups (for more information about *risk-based screening* see, e.g., Ref. [4]), unpredictability could become interesting when randomly checking passengers of the low-risk group to ensure a level of deterrence (see also the TSA's PreCheck program; [59]). However, it becomes apparent that an evaluation is necessary to draw further suggestions for practice.

### 4.2 Limitations and further research

A limitation of the present study is the rather small number of included experts. Although efforts have been made to involve as many experts as possible, it was difficult to reach the confidential exchange that we aimed for. One explanation is the topic itself, which is still regarded as security sensitive. As we used the network of our institutions to overcome this issue, we must consider that this approach could have also influenced our sample in terms of perspectives on the topic. Another explanation for the low number of experts willing to participate in the study might be that unpredictability measures are not (yet) the focus for most airports, and therefore the contribution of experts is aimed primarily at minimal compliance. Further, in regard to the topic of insider threats, the interviews showed that most experts are aware of it but perceive the topic as inconvenient because of the trust relationship between employer and employees.

With our sample of airports (one large and four small airports) we could not systematically investigate whether the importance of unpredictability depends on airport size. For example, one could argue that the security pattern in small airports is more predictable at some time and therefore, efforts to increase unpredictability could be more important at small airports. On the other hand, one could argue that the risk of insider threats is lower at small airports due to fewer security officers who know each other better than at large airports and therefore unpredictability measures targeting insider threats are less important for small airports. It would be interesting and valuable to continue and extend our research by systematically investigating which unpredictability measures are how important for small versus large airports.

As stated, our goal was to understand why and how unpredictability measures are applied. We did not aim for a comprehensive list of all applied applications. Instead, we tried to get detailed, rich information per respective measure for as many measures as possible. In other words, we prioritized quality before quantity which in consequence evokes the need for reflection in terms of resulted unpredictability measures. As we had limited time for the interviews with our experts, we chose to collect all applied measures in a first step before discussing some of them in more detail. In this phase, the role of the researcher was to navigate through the collected measures which of course influenced the final list in one way or another. During the data collection phase, the whole research team met regularly to reflect on collected material and to refine our approach. We agreed to manage a balance between gaining as much novel information (regarding measures) as possible and considering the experts’ view on the topic. For future research in this area, we recommend scheduling enough time to find, contact, and explain the study to potential interviewees. Researchers should also take into account that building trust between interviewer and expert on site takes time, especially when it comes to sensitive security information, which is why we would suggest, whenever possible, to start rather informally than begin immediately with the interview.

Furthermore, this study has shown that unpredictability measures are often not systematically evaluated regarding security effectiveness, operational efficiency, passenger experience, and deterrence. Further research could focus on the investigation of the deterrent effect of unpredictability measures, which in turn could help to improve a systematic evaluation of key performance indicators such as security effectiveness.

### 4.3 Conclusion

Unpredictability is an interesting approach in the field of airport security due to its potential for increasing effectiveness and efficiency of security controls. Although regulatory and practical efforts have been made to implement unpredictability in security systems at airports, no previous study has examined why and how unpredictability is implemented at airports. Our study addressed this research gap, and we have found that there are various reasons for applying unpredictability: Reasons which concentrate on complementing the security system, defeating the opponent, or on improving human factor aspects of the security system. When looking at the realization of unpredictability measures, various locations (where), controlling authorities (who) and forms of application (how) are already varied at airports, which opens a wide range of possibilities for future applications. Depending on several factors such as layout, infrastructure, or available (human) resources at a specific airport, different forms of application are conceivable. Results also show that unpredictability measures are used to address insider threats. The variation of a measure helps, for example, to reduce insider knowledge by increasing the non-transparency of the system and potentially increases their deterrent effect on insiders as well as outsiders. The implementation of unpredictability into existing security concepts is conceivable in a variety of ways: regular security measures could simply be varied (1), unpredictability measures could be added (2), or regular security measures could be replaced by unpredictability ones (3). From our perspective, an extended application, as suggested when replacing regular security measures, should include risk-based factors to differentiate high-risk from low-risk groups and maintain a high level of security. Future research should also focus on the evaluation of the deterrent effect of unpredictability to further give suggestions on how unpredictable measures should be realized to proactively defeat upcoming risks.

## Author contribution statement

Melina Zeballos: Conceived and designed the study; Performed the study; Analyzed and interpreted the data; Wrote the paper.

Carla Sophie Fumagalli: Performed the study; Analyzed and interpreted the data; Wrote the paper.

Signe Maria Ghelfi: Conceived and designed the study; Performed the study; Contributed materials, analysis tools or data; Wrote the paper.

Adrian Schwaninger: Conceived and designed the study; Contributed materials, analysis tools or data; Wrote the paper.

## Funding statement

This work was supported by Federal Office of Civil Aviation [BAZL/2016-138].

## Data availability statement

The data that has been used is confidential.

## Declaration of interest’s statement

The authors declare no conflict of interest.

## Figures and Tables

**Table:** 

Section	Topic	Questions
1	Concept of unpredictability	1. What do you [as an appropriate national authority/police organization/airline security manager/airport security manager] understand by the term «unpredictability»? 2. What comes to your mind when you think of unpredictability? 3. What benefits do you see in general concerning this concept? 4. What disadvantages do you see?
2	Application in general	5. What role/value does unpredictability play/have in general when applying/realizing security measures at the airport?
3	Overview of applied measures	6. What measures are already in use that you think have an unpredictability component? 7. What measures would you in particular name as unpredictability measures?
4	Implementation of applied measures	8. What: Description of the measure a. What kind of measure is it? b. Please describe (in brief) the measure … c. Is it an adapted or a new measure? d. Did the measure replace another measure or is it (an) additional (measure)? 9. Who: Initiation and implementation a. Who has initiated the measure? b. Who has implemented the measure in the end? (e.g., the police) c. Who is involved? 10. Where: Place of implementation at the airport a. Where was the measure applied/implemented? (e.g., Cargo) 11. Why: Reason/s of implementation of the measure a. Why was this measure introduced? (e.g., observations, incidents etc.) b. Did other measures also qualify for this purpose? If yes: How was the decision made for exactly this measure? 12. How: Concrete implementation of the measure a. How was this measure actually implemented? b. How often is this measure carried out? (frequency per unit time) c. How is this measure evaluated? d. What measured value (indicators) do you use? e. How is/was information passed about the measure (internal & external)? f. How do you communicate with employees about (new) unpredictability measures? 13. Lessons: Experiences (positive & negative) a. What experiences have you had with unpredictability measures? b. What are the challenges that have arisen during the implementation of (a) an unpredictability measure? How did you handle these challenges? c. Have you changed or optimized components of a measure or the measure itself? d. What would you do differently today? e. What are the positive experiences that you have had by implementing this measure? f. How do you plan to deal with the measure in the future? g. What experiences have you had regarding the type of communication? (internal & external)
5	Contribution to insider threats	14. How does your organization address the issue of insider threats? 15. In which areas (e.g., passenger screening) do you see a high risk of insider threats? 16. How has the importance of insider threats changed in recent years?
6	Future prospects of unpredictability	17. What is your final conclusion regarding unpredictability measures? 18. How do you assess the future development of unpredictability measures?
